# Preventive Dental Care Utilization in Asian Americans in Austin, Texas: Does Neighborhood Matter?

**DOI:** 10.3390/ijerph15102261

**Published:** 2018-10-16

**Authors:** Hyunwoo Yoon, Yuri Jang, Kwangyul Choi, Hyun Kim

**Affiliations:** 1 School of Social Work, The Texas State University, San Marcos, TX 78666, USA; 2 Edward R. Roybal Institute on Aging, Suzanne Dworak-Peck School of Social Work, University of Southern California, Los Angeles, CA 90007, USA; yurij@usc.edu; 3 Haskayne School of Business, Faculty of Environmental Design, University of Calgary, Calgary, AB T2N 1N4, Canada; kwangyul.choi1@ucalgary.ca; 4 Department of Geography, University of Tennessee, Knoxville, TN 37996, USA; hkim56@utk.edu

**Keywords:** neighborhood characteristics, preventive dental care utilization, Asian Americans

## Abstract

Although dental care is an essential component of comprehensive health care, a substantial proportion of the U.S. population lacks access to it. Disparities in dental care are most pronounced in racial/ethnic minority communities. Given the rapid population growth of Asian Americans, as well as the growing attention of neighborhood-level effects on health care use, the present study examines how individual-level variables (i.e., age, gender, marital status, ethnicity, education, place of birth, length of stay in the U.S., dental insurance, and self-rated oral health) and neighborhood-level variables (i.e., poverty level, density of Asian population, dentist availability, and Asian-related resources and services) contribute to predicting the use of preventive dental care in a sample of Asian Americans in Austin, TX. This study adds to the growing literature on the effect of neighborhood-level factors on health care as sources of disparities. Those living in the Census area with higher level of available dentists were more likely to use preventive dental care services. Findings suggest the importance of the location (proximity or accessibility) to dental clinics. In a planning perspective for health care policy, identifying the neighborhood with limited healthcare services could be a priority to diminish the disparity of the access.

## 1. Introduction

Although dental care is an essential component of comprehensive health care, a substantial proportion of the U.S. population lacks access to it [1,2,3]. According to the Medical Expenditure Panel Survey (MEPS), more than 2.7 million of the U.S. population reports that there was a time that they needed dental care but could not get it [4]. Disparities in dental care are more pronounced in racial/ethnic minority communities [5,6], and studies have consistently reported a lower rate of preventive dental care among African Americans and Hispanics compared to non-Hispanic Whites [7,8].

Asian Americans who have ancestral origins in any of the original populations of the Far East, South, Southeast Asia, or the Indian subcontinent are the fastest growing minority group and the largest group of new immigrants in the U.S. [9,10]. However, there is scarcity of information on this population’s oral health and dental care. Furthermore, findings from the existing studies are mixed. Studies based on population-based surveys, mostly using English as a primary survey language, portray Asian Americans favorably in terms of their access and utilization of dental care [4,11,12]. For example, Asian Americans had the lowest rate of unmet dental care needs among all racial/ethnic groups in the MEPS [4,12]. On the other hand, studies based on Asian American community ethnic samples present adverse status in oral health and dental care [13,14]. The seemingly contradictory findings could be attributed to a sampling bias where national surveys only included English-speaking Asian Americans. Given the fact that English-speaking Asian Americans are likely to be well acculturated individuals who have more education, higher income, and more favorable health status than those with limited English proficiency, findings based on English-proficient samples of Asian Americans are likely to be biased upward. Acknowledging the upward-selection bias in English-only national surveys, there is a compelling need to revisit the dental care issues in Asian Americans using a sample that reflect the population’s cultural and linguistic diversities.

At the individual-level, the guidelines for exploring factors associated with dental care in diverse populations have often been predisposing, need, and enabling variables of the Andersen’s behavioral health model [15]. Predisposing characteristics such as age and gender determine an individual’s propensity to use dental care. Both objective status and subjective perceptions of one’s oral health condition have been considered as a need for dental care [15,16,17]. Most attention has been paid to enabling variables that facilitate the use of dental care services. Dental insurance has been widely known as a critical enabler for dental service use [14,18,19,20]. For immigrant populations, factors that represent adaptations to a new country, such as length of residence, language proficiency, and acculturation, have shown to promote the use of dental care services [13,21,22].

Although the aforementioned studies on individual-level variables have improved our knowledgebase of dental care service use, environmental or contextual variables should also be taken into considerations. A growing body of literature suggests that the characteristics of the neighborhood in which people live have an impact on health and health service use [23,24,25]. As a Census-based neighborhood variable, poverty level has been commonly used, and studies demonstrate that individuals living within socioeconomically disadvantaged areas have poorer health and limited access to health care, especially when compared to their counterparts living in more advantaged areas [26]. Another important Census-based neighborhood level variable relevant to racial/ethnic minorities is the neighborhood composition of individuals with the same racial/ethnic background. Those residing in areas with high concentrations of people of the same ethnic background often report better health and service use outcomes than those living in areas with lower concentrations [27,28]. Given the regional variations in health service environments, it is also important to consider availability of medical services in one’s neighborhood. For example, a lack of availability of medical services in the area may pose health risks to its residents [26,29].

In addition to the customary use of Census-based neighborhood data, researchers have proposed the use of physical-environment data through direct community assessment [30,31]. This approach allows the use of a locally based definition of neighborhood and the ability to incorporate knowledge of local history and culture [32]. For example, direct assessment of ethnic communities (e.g., ethnically oriented services for food, shopping, religious activities, and health services) may lead to a greater understanding of how such resources, or the lack thereof, influence Asian Americans’ health and health care use. In the present study, a master list of ethnically oriented resources and services was generated, and each identified resource or service was geo-coded and used as one of neighborhood-level variables.

Taken together, the present study explored how individual-level variables (i.e., age, gender, marital status, ethnicity, education, place of birth, length of stay in the U.S., dental insurance, and self-rated oral health) and neighborhood-level variables (i.e., poverty level, density of Asian population, dentist availability, and Asian-related resources and services) contributed to predicting the use of preventive dental care in a sample of Asian Americans. We hypothesized that lower proportions of neighborhood residents living below the poverty level, higher proportions of Asian Americans, higher level of dentist availability, higher proportion of Asian-related resources and services would promote the use of preventive dental care services after the individual-level characteristics were controlled.

## 2. Methods

### 2.1. Dataset

#### 2.1.1. Individual-Level Data

Data were drawn from the Asian American Quality of Life (AAQoL) survey, which was part of the city of Austin’s initiative to improve response to the rapid growth of the Asian American population. An estimated 110,000 to 115,000 Asians live in metropolitan Austin, and the Asian community is doubling in size approximately every 12 years [33]. With approval from the University of Texas in Austin’s institutional review board (Protocol number: 2015-05-0015), survey data collection was conducted with Asian American residents in Austin, TX in 2015. The 10-page survey questionnaire was designed to address cultural and linguistic diversities. The questionnaire also required a residential address for survey participants, which allowed linkage between survey data and geographic information on neighborhood environment. The questionnaire was originally developed in English and then translated into the native languages of the five largest Asian subgroups living in Austin: Chinese (Chinese, in both traditional and simplified characters), Asian Indian (Hindi and Gujarati), Korean (Korean), Vietnamese (Vietnamese), and Filipino (Tagalog). The initial translations were conducted by eight professional translators and graduate level bilingual researchers. For each language, the translated version was reviewed and validated by two or more bilingual volunteers. Upon refinement of the questionnaire, each language version was pilot tested with three to five community members who spoke the target language, and their feedback was incorporated into the final version.

Self-identified Asians aged 18 and older living in the Austin area were eligible to participate. The research team contacted potential survey sites and made an arrangement for surveys. A total of 76 survey sessions took place at various sites across the city of Austin (i.e., churches, temples, grocery markets, small group meetings, and cultural events). The project was publicized through media and ethnic community sources, and referrals for individuals, groups, and organizations were actively sought. Self-administered surveys were conducted using a paper and pencil format with preferred language version. Bilingual research assistants were available at each survey site for recruitment and assistance with survey administration. It took about 20 min to complete the 10-page questionnaire, and all respondents were paid $10 for their participation. A total of 2614 individuals participated in the survey. After removing the 277 cases without a full residential address, the final sample size was 2337.

#### 2.1.2. Neighborhood-Level Data

To construct neighborhood level variables, Geographic Information Systems (GIS) served as a methodological tool. First, the reported residential addresses for survey participants were geo-coded to determine their Census Tract and then linked with Census and related data. Four variables in neighborhood-level (here in the Census Tract) were derived from the 2014 American Community Survey of the U.S. Census Bureau (e.g., poverty level and proportion of Asian American population), the Area Resource File by the Bureau of Health Professionals and Primary Care Service Area Files available from the U.S. Health Resources and Services Administration (e.g., dentist availability), and the direct community assessment (e.g., Asian-related resources and services). Thus, the data structure of the current study included participants’ survey data as well as newly constructed neighborhood/community characteristics for the Census Tract in which each participant resides.

### 2.2. Measures

#### 2.2.1. Individual-Level Variables

The use of preventive dental care services was measured by asking whether participants had visited dental clinics for a routine check-up in the past 12 months. Responses were coded as ‘no’ (0) or ‘yes’ (1).

Demographic information included age (in years), gender (0 = male, 1 = female), ethnicity (0 = Chinese, 1 = Asian Indian, 2 = Korean, 3 = Vietnamese, 4 = Filipino, 5 = Other Asian), marital status (0 = married, 1 = not married), education (in years), place of birth (0 = foreign born, 1 = U.S. born), and length of stay in the U.S. (in years). For dental health insurance coverage, participants were asked whether they had insurance that covered the cost of any dental visit. Responses were coded as ‘no’ (0) or ‘yes’ (1). Self-rated oral health was assessed by asking “How would you rate your overall oral health status?” The original five-point responses were dichotomized into ‘excellent/very good/good’ (0) and ‘fair/poor’ (1). The single-item measure has shown to be highly correlated with multiple-item subjective oral health measures and the results of clinical examination and often been used as a binary variable [16,17].

#### 2.2.2. Neighborhood-Level Variables

*Poverty level.* To evaluate a neighborhood’s economic conditions, we calculated the proportion of individuals living below the poverty level within each Census Tract group. This variable was *z*-transformed to have a range of 0 to 1, with higher scores representing a greater proportion of individuals living below the poverty level in the Census Tract.

*Asian American population.* As an indicator of population density of people with same racial background, the proportion of Asian Americans within the Census Tract was calculated. This variable was *z*-transformed to have a range of 0 to 1, with higher scores representing a higher proportion of Asian Americans living in the Census Tract. 

*Dentist availability.* Information on neighborhood dental service environments was extracted from the Area Resource File by the Bureau of Health Professionals and Primary Care Service Area Files available from the Health Resources and Services Administration. Proportion of dentists in the Census Tract to dentists in Austin was calculated by the total number of licensed-dentists in each Census Tract in which each participant resides divided by the total number of licensed-dentist in Austin Metropolitan Statistical Area. This variable was *z*-transformed to have a range of 0 to 1, with higher scores representing a higher level of availability of dental care services in the Census Tract. 

*Asian-related resources and services.* Along with AAQoL surveys, direct community assessment was conducted using a systematic method successfully used in previous research [31,34]. All available local resources and services offered primarily for Asians were included. Types of services included (1) places for medical care (e.g., doctor’s offices, dental practices, mental health services, pharmacies, centers of traditional medicine), (2) places for shopping and eating (e.g., groceries, restaurants), (3) places for religious activities (e.g., churches, temples), and (4) places for educational or leisure activities (e.g., senior centers, schools, clubs). Sources of data included web, yellow pages, Asian business directories, and local newspapers and other media. Two independent raters compiled a list of Asian community services and amenities, and then cross-assessments of the lists were conducted to maximize inclusion and check interrater agreement. Using street addresses, the identified resources and services were geo-coded, and the proportion of Asian-related resources and services within the Census Tract was calculated by the total number of Asian-related resources and services in each Census Tract in each participant resides divided by total number of Asian-related resources and services in the Greater Austin Metropolitan Area. It served as a measure of ethnic resource and service density in participants’ neighborhood. This variable was *z*-transformed to have a range of 0 to 1, with higher scores representing a higher proportion of Asian-related recourse and services in the Census Tract. 

### 2.3. Analytic Strategy

Descriptive analysis and bivariate correlations were conducted to assess the underlying structure of the study variables and to detect potential problems with collinearity. Because of the nested nature of the data (e.g., individuals within the Census Tract), we used the Multi-Level Model (MLM) to allow for the disentanglement of individual effects from contextual effects in the use of nested data [35]. The data structure of the current study included 2337 individual respondents (at level 1) nested within 226 Census Tracts (at level 2), with average 10.4 respondents within one Census Tract. Using a multilevel approach, we investigated the impact of several neighborhood characteristic on the use of preventive dental care services. Since the outcome variable (e.g., the use of preventive dental care services) is a dichotomous variable (0 = no, 1 = yes), we ran the two-level logistic regression model. Conceptually, the two-level logistic regression model is equivalent to the multilevel linear model except for the outcome variable.

The Level 1 model:

*y_ij_* ~ *Binominal* (1, π*_ij_*),

logit (π*_ij_*) = *β*_0*j*_ + *β*_1*j*_x*_ij_*

where *y_ij_* denote the binary response of interest (e.g., use of preventive dental care services) for individual respondent *i* in the Census Tract *j*, *β*_0_ is the intercept, and *β*_1_ is the coefficients of individual level covariates. In the level 1 model, characteristics of neighborhoods were not considered. 

The Level 2 model:

*β*_0*j*_ = *β*_0_ + *u_j_*

$$u_{j}\;\sim \;N\left(0,\;\sigma_{u}^{2}\right)$$

where *u_j_* denotes the random effect for the Census Tract *j. u_j_* is assumed to be normally distributed, with the expected zero mean and the variance $\sigma_{u}^{2}$. The level 2 model added the characteristics of neighborhood to the model in order to estimate the specific Odds Ratio for contextual variables. In the MLM analyses, Chinese served as a reference group because they are the largest and most studied Asian subgroup. The analyses of MLM were estimated using Mplus statistic software (Muthén and Muthén, Los Angeles, CA, USA).

## 3. Results

### 3.1. Descriptive Characteristics of the Participants and Their Neighborhood

Table 1 presents a summary of descriptive characteristics of the sample. The sample consisted of 2337 Asian American adults ranging in age from 18 to 98 years, with an average age of 42.8 (*SD* = 17.0). More than half (54.2%) were female, and 32.8% were unmarried. The sample included Chinese (24.3%), Asian Indians (23.2%), Koreans (18.8%), Vietnamese (18.0%), Filipinos (9.9%), and individuals from other Asian groups (5.8%). The ethnicities specified by participants in the ‘other’ group included Nepalese, Pakistani, Malaysian, Cambodian, and Japanese. Mean years in education is about 15 years, and about 83% had received more than a high school education. 9.4% of the sample were U.S.-born, and the length of stay in the U.S. ranged from 0.25 to 78 years, with an average of 15.4 (*SD* = 12.9). About 60% of the sample had dental insurance coverage, and 18.1% reported that their oral health was either ‘fair’ or ‘poor’.

With regard to neighborhood variables, the average proportion of individuals living below the poverty level in the current study was 0.14 (*SD* = 0.17). The average proportion of Asian Americans was 0.11 (*SD* = 0.08). The average proportion of dentists in the Census Tract to dentists in Austin was 0.003 (*SD* = 0.003). The average proportion of Asian-related resources and services in the Census Tract was about 0.005 (*SD* = 0.006).

### 3.2. Bivariate Correlations Among Study Variables

Bivariate correlations among study variables are summarized in Table 2. Advanced age, female, married status, being U.S.-born, longer stay in the U.S., having dental insurance, and positive self-ratings of oral health were correlated with use of preventive dental care services. With regard to neighborhood-level variables, lower proportions of individuals living below the poverty level, lower proportions of Asian Americans, higher level of available dentists, and less number of Asian-related resources and services were correlated with the use of preventive dental care services. All correlation coefficients were below 0.50, and the highest correlation was observed between proportions of individuals living below the poverty level and proportions of dentists in the Census Tract to dentists in Austin (*r* = −0.48, *p* < 0.001).

### 3.3. Multilevel Logistic Regression Models of the Use of Preventive Dental Care Services

The results of the multilevel analyses are presented in Table 3. At the individual-level, the odds of using preventive dental care services were higher among those who were female, stayed longer in the U.S., had dental insurance, and had positive self-ratings of oral health status. In comparison with the Chinese group, Asian Indian had higher odds for using preventive dental care services. At the neighborhood-level, living in the areas with a greater availability of dentists and with lower levels of Asian-related resources and services was associated with the increased odds of using preventive dental care services.

## 4. Discussion

Given the growth of Asian Americans [9,10] and the increased attention of neighborhood-level effects on health care use [36,37], the present study examined the effect of both individual- and neighborhood-level variables on the use of preventive dental care services among Asian Americans.

About 43% of the present samples did not use any preventive dental care services, which were higher than the rates (38%) reported in the national adult samples of Asian Americans [12]. Direct comparison of rates across different studies should be cautious due to heterogeneity of methodology, but this finding demonstrated the heightened vulnerabilities in preventive dental care access among Asian Americans.

As anticipated, dental insurance is the most critical enabler for the use of preventive dental care services at the individual-level, but a substantial proportion of Asian Americans in the current study lack such coverage. Such finding underscores the importance of increasing access to dental care for the uninsured and the underserved.

Moving beyond the effect of individual-level factors on preventive dental care use, this study sheds light on the role of neighborhood-level factors concerning the use of preventive dental care services. Lower proportions of individuals living below the poverty level were associated with the use of preventive dental care services at the bivariate level. This is consistent with findings from previous literature that those who reside in socioeconomically disadvantaged neighborhoods are likely to be deprived of socioenvironmental infrastructures or resources such as healthcare services [25,38]. However, the significance of the association disappeared in the multilevel analyses. One possible explanation for this finding may be that neighborhood poverty was itself associated with a cluster of both individual and neighborhood characteristics.

Lower proportions of Asian Americans were shown to be associated with the use of preventive dental care services at the bivariate level. This finding is contradictory to the previous findings that living in areas with high density of people with the same ethnic background led to better service use outcomes than their counterparts [27]. We also hypothesized that higher proportion of Asian-related resources and services would promote the use of preventive dental care services after controlling for the individual-level characteristics. However, it is worth noting that those living in a neighborhood with relatively abundant Asian-related resources and services tend to use less preventive dental care services. Given positive association between proportion of Asian population and proportion of Asian-related resources and services, a neighborhood with more Asian-related resources and services can be a desirable place to live for Asian Americans. However, aggregated neighborhood composition such as proportion of racial/ethnic minority population and of available ethnic resources and services may not influence individuals’ use of the preventive dental care services. Instead, such racial/ethnic composition and resources within neighborhoods should be combined with factors at the individual-level to predict the use of the preventive dental health care.

This study also adds to the growing literature on the effect of neighborhood-level factors on health care as sources of disparities. Those living in the Census Tract with higher level of available dentists were more likely to use preventive dental care services. Findings suggest the importance of the location (proximity or accessibility) to dental clinics. In a planning perspective for health care policy, identifying the neighborhood with limited healthcare services could be a priority to diminish the disparity of the access.

Some limitations to the present study should be noted. The present study used a regionally defined sample and Census Tract level analysis. The effects of neighborhood characteristics may vary in other cities and states with different spatial units for analysis. Although a special effort was made to represent diversities of the Asian American population in Austin, TX, the current study is based on a sample of convenience. The use of a non-representative sample and a cross-sectional design limits causal inferences and generalization of the findings to the larger population of Asian Americans. Future studies should revisit the topic with a representative sample and longitudinal design. Although the estimated models considered multiple key factors at both individual- and neighborhood-level, future efforts should incorporate a broader range of variables, including type of dental health insurance coverage, individual’s attitude toward dental care use, acculturation, the availability and accessibility of culturally and linguistically competent dental care professionals in local dental service environments, as well as residents’ perception and response to their environment. This may improve understanding of how neighborhood factors influence Asian Americans access to dental care. The findings of the current study provided baseline information on public health policies and programs for Asian Americans as an aggregated group, but given the heterogeneity of Asian American populations, future studies should be taken into consideration within-group variation in Asian American population. Consideration of differences in acculturation based upon nativity or the length of stay in the U.S. among different ethnic groups would help develop preventive dental care services and programs tailored to meet unique needs of the target ethnic group. 

## 5. Conclusions

Despite these limitations, the present study sheds light on the importance of using culturally and linguistically sensitive approaches (e.g., Asian language versions of the survey questionnaire and research personnel who shared the languages and cultures of the target population) to reach out to the Asian-American population. Furthermore, this study expands understanding of the use of preventive dental health care by identifying neighborhood-level factors as a possible determinant and source of disparities.

## Figures and Tables

**Table 1:** Descriptive characteristics of the sample (*n* = 2337).

Variables	M ± SD (Range) or %
***Individual level-variables***	
Age (years)	42.8 ± 17.0 (18–98)
Gender (female)	54.2
Marital status (unmarried)	32.8
Ethnicity	
Chinese	24.3
Asian Indian	23.2
Korean	18.8
Vietnamese	18.0
Filipino	9.9
Other	5.8
Education (years)	14.9 ± 3.50 (0–17)
Place of birth (U.S. born)	9.4
Length of stay in the U.S. (years)	15.4 ± 12.9 (0–78)
Dental insurance (yes)	59.9
Self-rated oral health (fair/poor)	18.1
***Neighborhood-level variables***	
Poverty level	0.14 ± 0.17 (0–0.84)
Asian American population	0.11 ± 0.08 (0–0.34)
Dentist availability	0.003 ± 0.003 (0–0.03)
Asian-related resources and services	0.005 ± 0.006 (0–0.05)
***Outcome variable***	
Use of the preventive dental care services (yes)	57.2

**Table 2:** Correlations Among Study Variables.

Variable	1	2	3	4	5	6	7	8	9	10	11	12	13	14
1. Age	-													
2. Gender	0.04	-												
3. Marital status	−0.41 ***	−0.01	-											
4. Ethnicity	0.05 *	0.07 **	0.04 *	-										
5. Education	−0.16 ***	−0.15 ***	−0.13 ***	−0.30 ***	-									
6. Place of birth	−0.31 ***	−0.01	0.28 ***	0.07 ***	−0.13 ***	-								
7. Length of stay in the U.S.	0.43 ***	0.04 *	−0.06 **	0.16 ***	−0.17 ***	0.25 ***	-							
8. Dental insurance	−0.09 ***	0.01	−0.13 ***	0.04	0.22 ***	0.94 ***	0.15 ***	-						
9. Self−rate oral health	0.29 ***	0.01	−0.03	−0.09	−0.16 ***	−0.18 ***	0.07 **	−0.18 ***	-					
10. Poverty level	−0.26 ***	−0.05 *	0.28 ***	0.13 ***	−0.17 ***	0.12 ***	−0.09 ***	−0.13 ***	−0.04	-				
11. Asian American population	−0.11 ***	−0.03	−0.02	−0.15 ***	0.06 **	0.00	−0.15 ***	0.02	−0.04	−0.02	-			
12. Dentist availability	0.07 **	0.03	−0.12 ***	−0.20 ***	0.19 ***	−0.07 **	−0.03	0.06 **	−0.02	−0.48 ***	0.05 **	-		
13. Asian−related resources and services	−0.20 ***	−0.02	0.12 ***	−0.02	0.00	0.09 ***	−0.15 ***	−0.04 *	−0.05 *	0.22 ***	0.32 ***	0.11 ***	-	
14. Use of the preventive dental care services	0.10 ***	0.10 ***	−0.07 ***	0.04	0.04	0.07 ***	0.31 ***	0.39 ***	−0.12 ***	−0.11 ***	−0.04 **	−0.07 **	0.06 **	-

* *p* < 0.05. ** *p* < 0.01. *** *p* < 0.001.

**Table 3:** Multilevel logistic regression model of the use of preventive dental care services.

Variables	OR	95% CI
***Individual-level***		
Age (years)	1.01	0.99–1.02
Gender (Reference: male)		
Female	1.52 ***	1.25–1.86
Marital status (Reference: married)		
Unmarried	1.10	0.87–1.40
Ethnicity (Reference: Chinese)		
Asian Indian	1.78 ***	1.12–2.82
Korean	0.72	0.46–1.14
Vietnamese	1.11	0.70–1.77
Filipino	1.37	0.85–2.21
Other	1.38	0.82–2.34
Education (years)	1.01	0.96–1.06
Place of birth (reference: foreign born)		
U.S. born	1.14	0.75–1.73
Length of stay in the U.S. (years)	1.04 ***	1.03–1.05
Dental insurance (Reference: no)		
Yes	5.44 ***	4.38–6.77
Self-rated oral health (Reference: excellent/very good/good)		
Fair/poor	0.78 ***	0.71–0.87
***Neighborhood-level***		
Poverty level	1.01	0.99–1.01
Asian American population	0.99	0.98–1.01
Dentist availability	1.42 *	1.02–1.98
Asian-related resources and services	0.82 **	0.72–0.93
Akaike (AIC)	2465.6	
Bayesian (BIC)	2585.2	

* *p* < 0.05. ** *p* < 0.01. *** *p* < 0.001.
